# Mismatch Between Cancer Burden and Clinical Trial Activity in the European Union: Analysis of Incidence, per Capita Indicators and Commercial Versus Non-Commercial Studies

**DOI:** 10.3390/cancers18142347

**Published:** 2026-07-21

**Authors:** Klaudia Marciniak, Maja Janowczyk, Michał Brancewicz, Marlena Robakowska

**Affiliations:** 1 Faculty of Health Sciences with the Institute of Maritime and Tropical Medicine, Medical University of Gdańsk, 80-210 Gdańsk, Poland; marciniak789@gumed.edu.pl (K.M.); maja.janowczyk@gumed.edu.pl (M.J.); 2 Department of Public Health & Social Medicine, Medical University of Gdańsk, 80-210 Gdańsk, Poland; mrobakowska@gumed.edu.pl

**Keywords:** cancer burden, clinical trial activity, European Union, research mismatch, commercial research, non-commercial research

## Abstract

**Introduction:** Cancer represents one of the greatest public health challenges in Europe. Clinical trials play a key role in the development of new diagnostic and treatment methods, but their geographic distribution may not reflect the true epidemiological burden. The aim of this study was to assess the relationship between the incidence of selected cancers and clinical trial activity in European Union countries, with particular emphasis on commercial and non-commercial trials, as well as changes over time. **Materials and Methods:** Data on clinical trials conducted in European Union countries in 2012, 2014, 2017, and 2022 were analyzed. Epidemiological data were obtained from the GLOBOCAN and Global Burden of Disease databases, while clinical trial data were obtained from the EU Clinical Trials Register. Overall cancer incidence and selected cancer types, namely breast cancer, lung cancer, colorectal cancer, and prostate cancer, were analyzed. Clinical trial activity rates were calculated relative to incidence and population size (per capita). Pearson and Spearman correlation analyses were performed, and 95% confidence intervals and *p*-values were calculated for the principal correlations. Time trends were also assessed. The results are presented in the form of tables, figures, and maps. **Results:** Clinical trial activity in oncology varied considerably across European Union countries. Commercial trials were consistently more common than non-commercial trials and showed a more pronounced increase in activity during the analyzed period. Correlations between cancer incidence and the number of clinical trials varied depending on cancer type, year of analysis, and sponsor type. The strongest associations were observed in 2022 for commercial trials, particularly for colorectal cancer (Pearson’s r = 0.865, 95% CI: 0.723–0.937, *p* < 0.001) and lung cancer (Pearson’s r = 0.853, 95% CI: 0.701–0.931, *p* < 0.001). Spearman analysis confirmed strong rank-based associations in 2022, especially for lung cancer (ρ = 0.971, 95% CI: 0.937–0.987, *p* < 0.001). Analysis of trial activity indicators indicated a mismatch between disease burden and the intensity of research activity in some countries and oncology areas. **Conclusions:** Clinical trial activity in oncology in European Union countries does not always reflect the actual cancer burden. The observed differences between commercial and non-commercial trials and between individual cancer types may indicate a research gap and the need for more balanced clinical trial planning in the European Union.

## Simple Summary

Cancer is a major health problem in Europe, but clinical trials are not always distributed according to where the need is greatest. This study examined whether the number of oncology clinical trials in European Union countries reflects the burden of cancer in those countries. We compared cancer incidence with clinical trial activity for selected cancer types, includingbreast, lung, colorectal, and prostate cancer, and assessed differences between commercial and non-commercial trials over time. The findings show that clinical trial activity varies considerably between countries and cancer types and that commercial trials are more common than non-commercial trials. In some areas, the number of trials does not appear to match the burden of disease. These results may help researchers, policymakers, and institutions better understand gaps in clinical trial availability and support more balanced planning of cancer research across Europe.

## 1. Introduction

Cancer represents a disproportionately large health burden in Europe, which comprises less than 10% of the world’s population yet accounts for over 20% of global deaths and over 23% of cancer diagnoses. In the European Union, approximately 2.6 million new cases of cancer are diagnosed each year, and approximately 1.2 million people die from it [1]. The most common cancers include breast, prostate, lung, and colorectal cancers, which together account for approximately half of all new cases [2].

Cancer prevention and treatment have become one of the public health challenges of the 21st century [3]. Clinical trials are a key aspect in this regard. They play a crucial role in the development of new treatments and in improving the outcomes of existing therapies.

However, access to modern therapies and cancer-related healthcare services in the European Union remains highly variable and heterogeneous. Broader inequalities across the cancer pathway have been documented in Europe, including differences in cancer risk factors, screening, early diagnosis and outcomes across and within countries [4]. In addition, previous reports have highlighted that access to cancer clinical trials in the European Union may be limited by geographic, clinical and regulatory barriers [5]. As a result, the availability and distribution of clinical trials do not always reflect the actual disease burden in individual regions and countries [6].

The distinction between commercial and non-commercial research is also important in this context. These types of studies differ in their funding sources, research goals and organizational conditions, which may influence the direction of research development and contribute to a mismatch with the actual health needs of the population [7]. In addition, previous reports have suggested that regulatory and administrative requirements may pose challenges for non-commercial oncology trials in Europe [8].

Despite the growing number of clinical trials in oncology and access to epidemiological data, existing studies do not provide a systematic assessment of the alignment of research activity with the actual disease burden in European Union countries. In oncology, global analyses have also shown that cancer clinical trials are unevenly distributed across countries and regions, raising concerns about the alignment between research activity and population health needs [9]. Previous studies have shown that access to oncology clinical trials is highly uneven across and within countries, highlighting the need for analyses linking trial activity with population-level cancer burden [10].

In particular, there is a lack of analyses that simultaneously consider time trends, the incidence of specific cancer types, and the distinction between commercial and non-commercial trials.

Consequently, it remains unclear whether oncology research activity in EU countries meets actual epidemiological needs and whether there is a mismatch between incidence and the number of trials conducted. This issue is also relevant in the context of recent European policy discussions calling for more strategic, coordinated and impact-oriented investment in cancer research across Europe [11].

This study aims to assess the correspondence between cancer burden and clinical trial activity in EU countries, with particular emphasis on differences between commercial and non-commercial trials and time trends. The study focuses particularly on the most common cancer types: breast, prostate, lung, and colorectal cancer. Clinical trial activity does not appear to proportionally reflect the cancer burden.

This study fills a significant gap in the literature by providing systematic data on the alignment of research activity with epidemiological needs in the context of European Union countries. This could support public health initiatives and improve the organization of clinical trials and cancer care.

## 2. Methods

This study is a retrospective observational analysis of clinical trial activity in oncology in European Union countries. The study focused on European Union countries due to their shared regulatory framework for clinical trials and relatively comparable healthcare systems, which facilitates more consistent and reliable cross-country analysis. Cancer incidence data were obtained from the Global Cancer Observatory (GCO) database [3]. For countries with missing epidemiological data, including Belgium, Bulgaria, Greece, Spain, Luxembourg, Portugal, Romania, Slovakia, and Hungary, alternative data from the Global Burden of Disease (GBD) database were used [12]. However, these data are limited to specific disease entities and do not provide overall cancer incidence.

Clinical trial information was obtained from the public Clinical Trial Information System (CTIS) website, accessible through the European Medicines Agency’s Clinical Trials in the European Union portal [13]. A search was conducted for interventional clinical trials in oncology conducted in European Union Member States. Trials were identified based on keywords corresponding to the names of the cancers analyzed. The disease entities identified corresponded to the ICD categories used in the analysis. In the case of older trials, the database may have included trials originally registered in the EudraCT/EU Clinical Trials Register and subsequently made available in CTIS due to the transitional period for implementing Regulation (EU) No 536/2014 [14]. Multicenter trials conducted in more than one country were counted separately for each participating country. Therefore, the data presented reflect the level of participation in clinical trials by country rather than the number of unique trials, which may lead to an overestimation of research activity in countries that participate more frequently in international projects. Population data were obtained from Eurostat [15].

**Figure 1:**
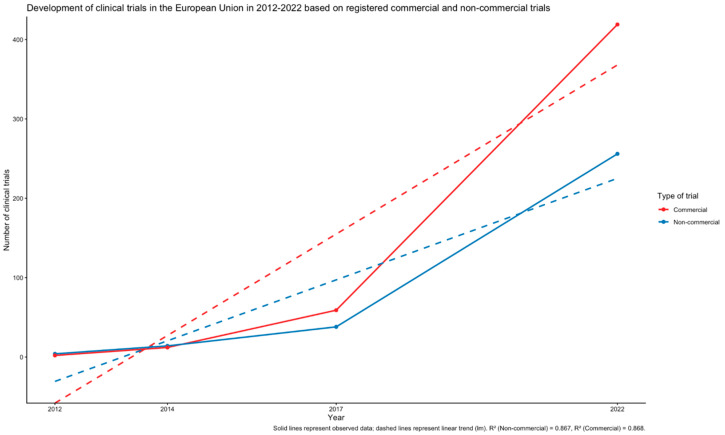
Development of clinical trials in the European Union in 2012–2022 based on registered commercial and non-commercial trials.

The year 2022 was selected as the primary reference year because it is the most recent year for which complete and comparable epidemiological data are available in the GCO database. To assess changes over time, data from 2017 and 2014 were included in all comparative analyses. Data from 2012 were used only for the descriptive presentation of clinical trial activity and for the temporal trend analysis (Figure 1), as the number of registered clinical trials in that year was very limited. This limited availability may be related to the transition and harmonization of the European clinical trial registration system following Regulation (EU) No 536/2014 of the European Parliament and of the Council. Therefore, 2012 was not included in the correlation analyses or the activity rate analyses.

The analysis covered all EU countries included in the GCO study. The study focused on the incidence of all cancers and differentiated them into the most common types: breast cancer (C50), lung cancer (C33–C34), colorectal cancer (C18–C21), and prostate cancer (C61). Lung cancer includes neoplastic lesions in the trachea, bronchi and lungs. Data included individuals of both sexes and ages 0–85+, allowing for the full range of both adult and pediatric populations to be included in the epidemiological analysis.

To summarize the collected data, they were tabulated, and descriptive statistics were used. The relationship between the number of cancer cases and clinical research activity was assessed using Pearson and Spearman correlation analyses. Pearson’s correlation coefficient was used as the primary measure of linear association between numerical values. Spearman’s rank correlation coefficient was used as a rank-based sensitivity analysis, allowing for the assessment of the consistency of the ranking of countries according to disease burden and clinical research activity, without assuming a linear relationship and with reduced sensitivity to outliers. For Pearson correlations, 95% confidence intervals and *p*-values were calculated using standard correlation testing. For Spearman correlations, *p*-values were calculated using Spearman’s rank correlation test, and 95% confidence intervals were estimated based on the correlation of ranked variables. A *p*-value of <0.05 was considered statistically significant. Data organization and preliminary tabulation were performed using Microsoft Excel 365. All statistical analyses were performed using R software, version 4.6.0 (R Foundation for Statistical Computing, Vienna, Austria), in RStudio, version 2026.1.2.418 (Posit Software, PBC, Boston, MA, USA). The mismatch between disease burden and clinical research activity was assessed using the clinical research activity-to-incidence ratio, defined as the number of clinical trials per 100,000 new cancer cases and was calculated according to the formula: number of clinical trials/numbers of new cancer cases × 100,000. This ratio was used to identify countries with relatively higher or lower clinical research activity relative to cancer burden. Analyses were performed separately for commercial and non-commercial trials and for individual cancer types, and the results were presented in tabular and graphical formats. Importantly, separate *y*-axis scales were applied for each year in the bar charts to better illustrate country-level variation within a given year. Therefore, the visual height of the bars should not be directly compared between years; comparisons over time should be based on the numerical values shown in the figures and the reported value ranges. To account for differences in population size between countries, population-adjusted rates—study per capita were also calculated. The number of clinical trials per 1 million inhabitants was used as a measure of research activity and was calculated according to the formula: number of clinical trials/population × 1,000,000. Geographic visualization using a map of Europe was used to present the results of this analysis. Importantly, color scales were adjusted separately for each year to better illustrate spatial variation within a given year. Color intensities should not be directly compared between years. The analysis of research activity rates and per capita rates did not include countries for which data on overall cancer incidence were missing (Belgium, Bulgaria, Greece, Hungary, Luxembourg, Portugal, Romania, Slovakia, and Spain).

Temporal trends were evaluated using plots, including trend lines and the coefficient of determination (R^2^). Unlike the country-level analyses, where multicenter studies were counted separately for each participating country, the time-trend analysis included only unique clinical trials across the European Union. Therefore, each study was counted once, regardless of the number of participating countries.

This analysis relies on heterogeneous data sources, which may affect comparability. Therefore, the results should be interpreted as exploratory.

## 3. Results

The country-level data on cancer incidence and oncology clinical trial activity are presented in Table 1, Table 2, Table 3 and Table 4. Table 1 shows the data for 2022, while Table 2, Table 3 and Table 4 present the data for 2017, 2014 and 2012, respectively. Correlation analyses and activity rate analyses were performed for 2014, 2017, and 2022 only. Data from 2012 were included exclusively in the descriptive summary (Table 4) and the temporal trend analysis (Figure 1).

The total number of cancer cases in 2022 in the 27 European Union countries was 3,293,731, with the highest incidence in the Czech Republic, Germany, France, and Italy. Conversely, when it comes to oncological testing for residents in EU countries, the median number of non-commercial trials (4) was lower than the median number of commercial trials (64). Commercial trials clearly dominated in all analyzed years, with median values significantly higher than non-commercial trials. Most studies published in 2022 were conducted in Spain, France, and Italy. Non-commercial trials were also conducted in France, Italy, and Spain. Additional variation was also observed between clinical trials for different cancer types, with the number of scientific trials ranging from 0 to 132 and non-commercial trials from 0 to 22 across EU countries. There was a general increase in the number of clinical trials over time, particularly between 2017 and 2022.

Figure 1 shows the growth in the number of clinical trials, both commercial and non-commercial. Commercial trials increased significantly from 12 in 2014 to 419 in 2022. Non-commercial trials also saw a growth, but at a smaller rate, from 14 in 2014 to 256 in 2022. Trend analysis shows a steady upward trend.

As shown in Table 5, the strongest and most consistent correlations between cancer incidence and clinical trial activity were observed in 2022, particularly for commercial trials. For commercial trials, strong positive Pearson correlations were found for colorectal cancer (r = 0.865, 95% CI: 0.723–0.937, *p* < 0.001), lung cancer (r = 0.853, 95% CI: 0.701–0.931, *p* < 0.001), prostate cancer (r = 0.771, 95% CI: 0.552–0.890, *p* < 0.001), and breast cancer (r = 0.760, 95% CI: 0.534–0.885, *p* < 0.001). Spearman analysis confirmed strong rank-based associations in 2022, especially for lung cancer (ρ = 0.971, 95% CI: 0.937–0.987, *p* < 0.001), breast cancer (ρ = 0.929, 95% CI: 0.848–0.967, *p* < 0.001), and colorectal cancer (ρ = 0.902, 95% CI: 0.794–0.955, *p* < 0.001). In previous years, particularly in 2017 and 2014, the associations were weaker and less consistent. In 2017, statistically significant positive Pearson correlations were observed for selected cancer types, including breast cancer in commercial trials (r = 0.528, 95% CI: 0.185–0.756, *p* = 0.005), lung cancer in commercial trials (r = 0.519, 95% CI: 0.174–0.751, *p* = 0.005), and prostate cancer in commercial trials (r = 0.525, 95% CI: 0.181–0.754, *p* = 0.005), whereas colorectal cancer showed weak and non-significant correlations. In 2014, significant positive Pearson correlations were observed for breast cancer in commercial trials (r = 0.546, 95% CI: 0.210–0.767, *p* = 0.003) and lung cancer in commercial trials (r = 0.584, 95% CI: 0.263–0.789, *p* = 0.001). Overall, these results indicate that the relationship between cancer incidence and clinical trial activity became stronger and more consistent in later years, particularly for commercial trials.

**Figure 2:**
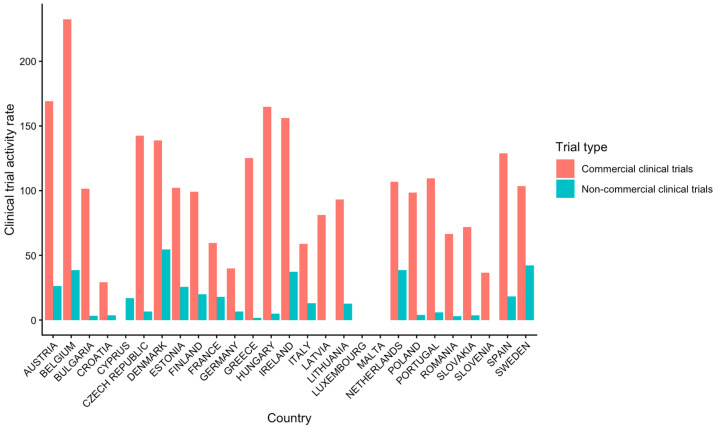
Clinical research activity-to-incidence ratio in European Union countries in 2022.

**Figure 3:**
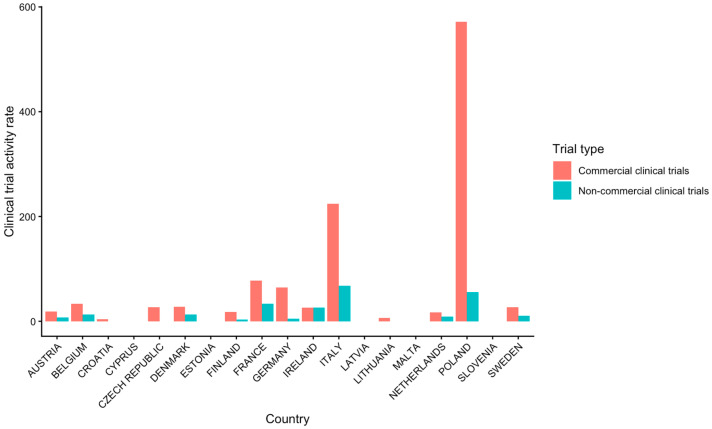
Clinical research activity-to-incidence ratio in European Union countries in 2017.

**Figure 4:**
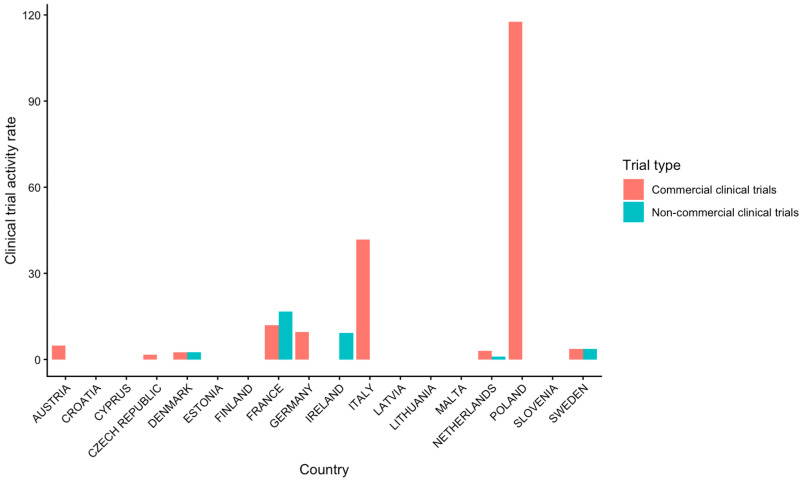
Clinical research activity-to-incidence ratio in European Union countries in 2014.

Clinical trial activity in EU countries relative to the overall cancer incidence is shown in Figure 2, Figure 3 and Figure 4. The ratio of non-commercial clinical trials to cancer cases varied significantly between countries, ranging from 0 to 54.6 for 2022, from 0 to 67.9 in 2017, and from 0 to 16.6 in 2014. In contrast, the ratio of commercial clinical trials ranged from 0 to 232.5 for 2022, from 0 to 571.8 for 2017, and from 0 to 117.7 for 2014. In 2022, the highest rate of research activity in commercial trials was observed in Belgium. The same rate in non-commercial trials was observed in Denmark. In 2017, the highest rate in commercial research was observed in Poland, while the highest rate in non-commercial research was observed in Italy. In 2014 the highest rate in commercial research continued to be recorded in Poland, while the highest rate in non-commercial research was recorded in France.

**Figure 5:**
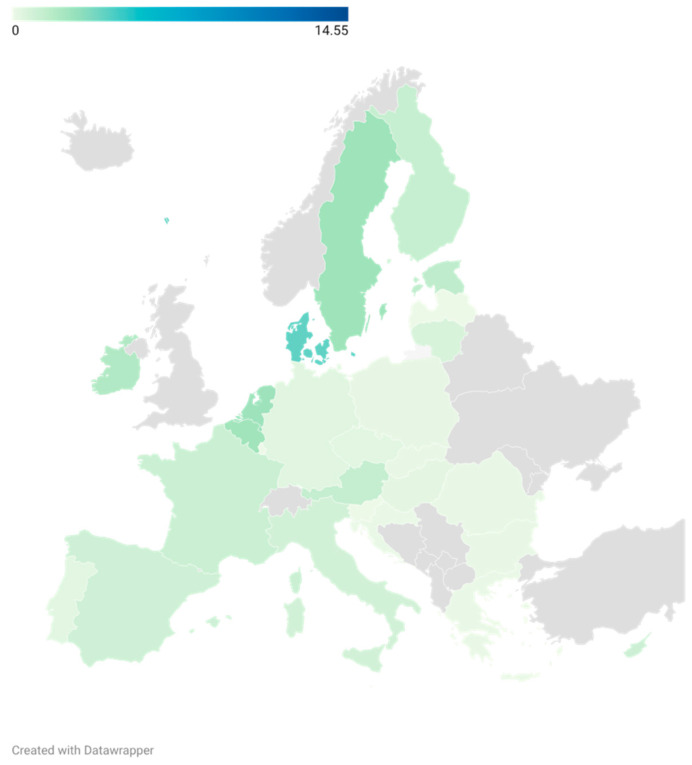
Geographic distribution of per capita non-commercial clinical trial activity across European Union countries in 2022.

**Figure 6:**
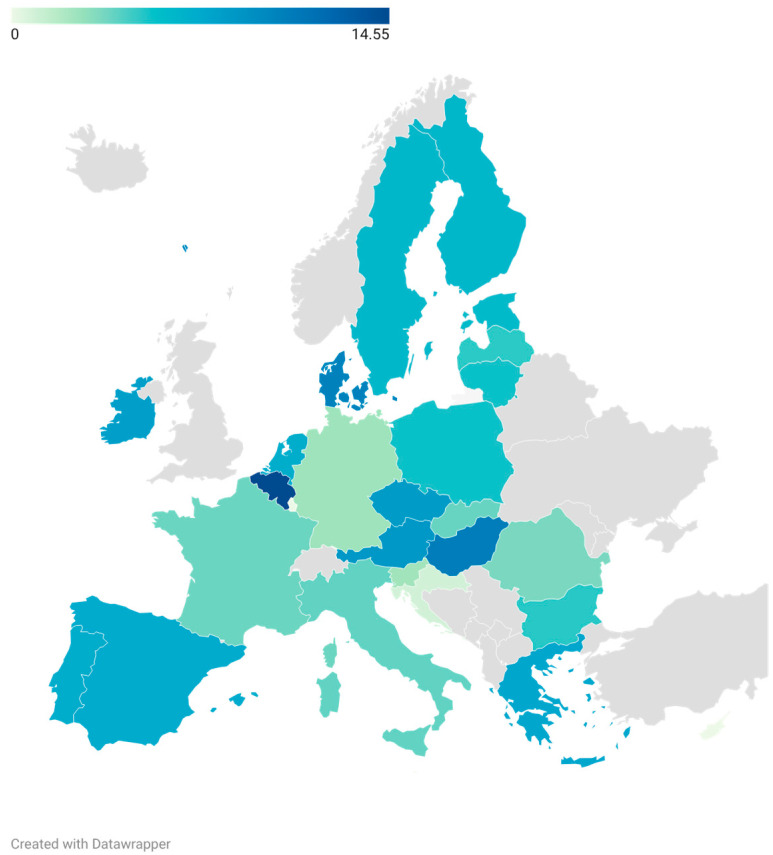
Geographic distribution of per capita commercial clinical trial activity across European Union countries in 2022.

**Figure 7:**
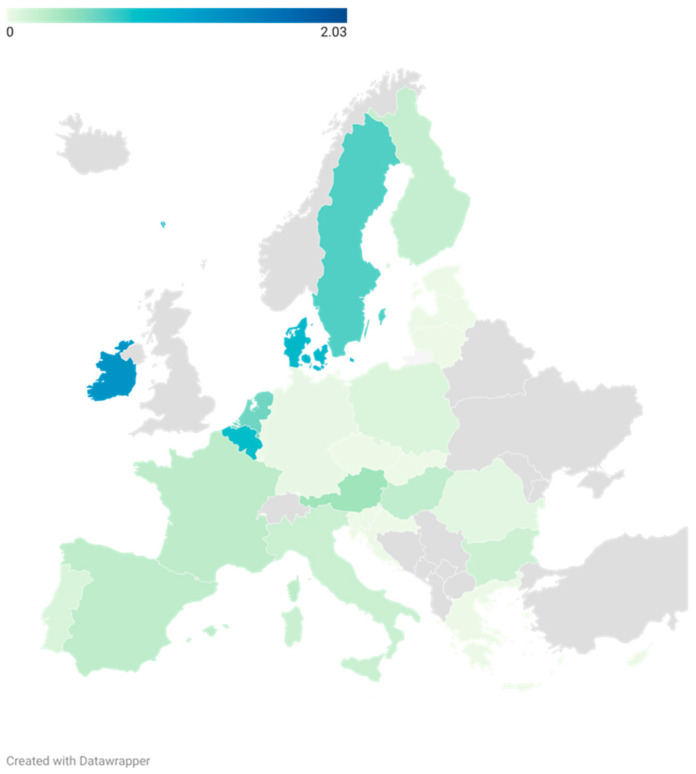
Geographic distribution of per capita non-commercial clinical trial activity across European Union countries in 2017.

**Figure 8:**
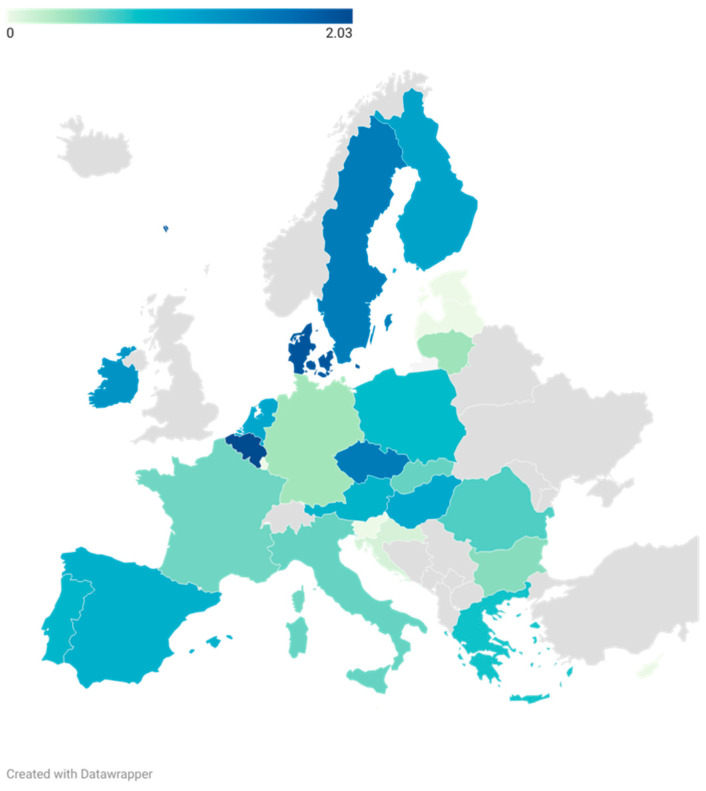
Geographic distribution of per capita commercial clinical trial activity across European Union countries in 2017.

**Figure 9:**
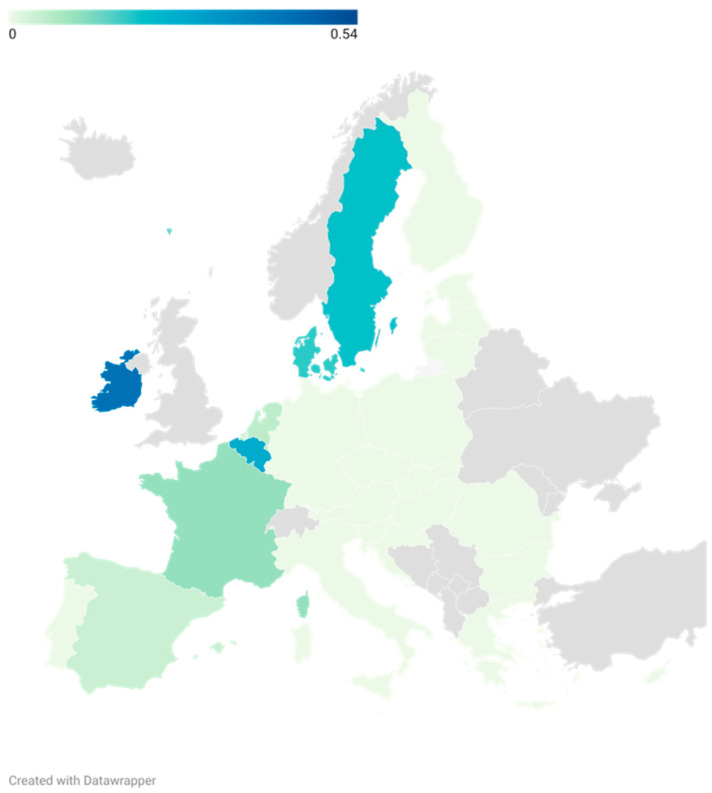
Geographic distribution of per capita non-commercial clinical trial activity across European Union countries in 2014.

**Figure 10:**
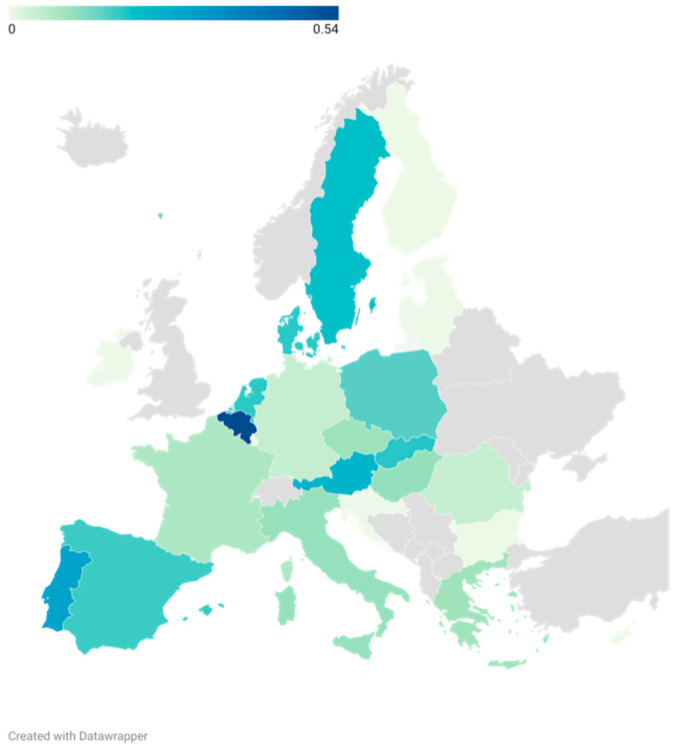
Geographic distribution of per capita commercial clinical trial activity across European Union countries in 2014.

The clinical trial activity rate, defined as the number of clinical trials per 1 million inhabitants in each country, is presented in Figure 5, Figure 6, Figure 7, Figure 8, Figure 9 and Figure 10. After considering population size, the differences between countries became more pronounced, with per capita values for non-commercial research ranging from 0 to 4 for 2022, from 0 to 1.2 for 2017, and from 0 to 0.4 for 2014. For commercial research, per capita values ranged from 0 to 14.5 for 2022, from 0 to 2 for 2017, and from 0 to 0.5 for 2014. In 2022, the same countries in the same areas consistently had the highest per capita research activity rates (Belgium: commercial and Denmark: non-commercial). For both 2014 and 2017, the highest research activity in commercial trials was observed in Belgium and in non-commercial trials in Ireland.

Both incidence-adjusted and population-adjusted rates revealed significant heterogeneity in clinical trial activity across countries.

## 4. Discussion

This study provides a comprehensive, retrospective comparative analysis of clinical research activity in oncology compared to disease burden in European Union countries. The results demonstrate significant heterogeneity between countries, a clear dominance of commercial trials, and a general upward trend in the morbidity-research activity ratio over a 10-year period.

One of the most important findings is the significant imbalance between commercial and non-commercial research. Commercial trials consistently outnumbered non-commercial trials in all analyzed years in European Union countries. The predominance of commercial trials observed in this study is consistent with previous reports highlighting the significant role of industry sponsorship in shaping clinical research activity [16,17]. Industry-sponsored clinical trials have a significantly higher globalization rate than non-industry-sponsored trials, with 30% of industry-led clinical trials being conducted in preferentially selected countries [18]. This trend demonstrates that research activity is at least partially driven by market considerations rather than solely by public health needs.

Another important observation is the improvement in the correlation between cancer incidence and clinical trial activity over time. While these correlations were weak or negligiblein previous years, in the 2022 analyses the strength of this relationship increased, suggesting a better alignment of disease burden with research efforts. This trend may reflect a growing awareness of the need to align research priorities with epidemiological data, as well as improvements in data availability and trial registration systems [19,20]. The consistency of the Pearson and Spearman correlation results supports the credibility of the observed relationships and suggests that they were not solely due to extreme values or the assumption of a strictly linear relationship. At the same time, these results should be interpreted with caution—they indicate the coexistence of a higher disease burden and greater research activity but do not constitute evidence of a full adjustment of the number of clinical trials to the actual epidemiological needs.

Despite this improvement, there was evidence of a discrepancy between cancer burden and the distribution of clinical trials, particularly in previous years (2017 and 2014). In some cases, countries with high incidence rates showed relatively low research activity, suggesting potential mismatched representation in clinical trials. Conversely, some countries showed disproportionately high research activity relative to their disease burden. These findings highlight persistent inequalities in the distribution of clinical trials across the European Union. Recent global analyses also indicate that geographical disparities in oncology trial distribution and access persist despite the overall growth in clinical trial activity [21].

Additionally, the per capita analysis revealed disparities, highlighting differences in research intensity that were not fully apparent using absolute numbers alone. This analysis suggests that a population-adjusted indicator provides a more accurate picture and should be used in future analyses of this topic.

The observed patterns, consistent with previous research by Hadrien Moffroid [22], reveal that clinical research activity is often concentrated in regions with stronger funding, industry, and infrastructure. Our results reflect this. A higher number of commercial trials was observed in countries such as Spain, France, and Italy, which are characterized by high levels of economic development. Furthermore, the predominance of commercial research may contribute to a focus on certain cancer types that are more attractive from a market perspective, potentially leading to underrepresentation of other disease areas [23].

Academic and non-commercial research focuses on optimizing existing therapies, analyzing drug combinations, and tailoring treatment to patient needs, even after drug launch. In many cases, these studies form the basis of oncological treatment standards [24]. As noted by Negrouk and her co-authors academic clinical trials conducted in Europe have significantly contributed to the development of effective cancer treatments. More recent European initiatives have also emphasized that independent academic cancer trials are vital for improving patient outcomes worldwide, as they often address clinically relevant questions that are not necessarily prioritized by commercial sponsors [25]. Therefore, it is worthwhile to focus on and develop non-commercial research, as these studies play a significant role in protecting public health by, for example, improving the quality of care and safety. They allow for tailoring therapy to the actual needs of the population. However, conducting international clinical trials can still pose a significant challenge for academic sponsors [26,27]. However, their implementation is possible thanks to appropriate mechanisms for governance, financing, and close collaboration with international research groups and, in some cases, with the industry. Marta del Álamo and her co-authors proposes several solutions to this problem: for example, public funding should be prioritized and targeted at the goals of international non-commercial clinical trials. Next, flexible cross-border financing mechanisms should be implemented, and the development of clinical research infrastructure and networks should be maintained [28]. Despite progress in international cooperation, cross-border funding for non-commercial research remains a challenge, primarily due to administrative constraints and limited funding [29]. Increasing the volume of non-commercial research requires, above all, increased public funding and long-term grants that allow for research without profit pressures. A change in the system of researcher evaluation is also crucial—we need to increase the emphasis on the quality of knowledge and scientific impact instead of commercialization. Open science, international collaboration, and support from independent foundations are also important, as they can significantly boost the development of non-commercial research. Several organizations offer partnerships and funding for non-commercial research, but the demand for them is constantly growing. It should also be emphasized that annual research investment under Horizon 2020, which aims to support international collaborative research initiatives, represents only about 4% of the total R&D investment of EU Member States [24]. This is still insufficient to effectively increase the volume of non-commercial research in Europe.

### Limitations

This study has several limitations that should be considered when interpreting the results. First, data from different sources (GCO, GBD, EUCTR) were used, which differ in methodology and reporting standards, which may affect their comparability. Future studies should incorporate harmonized data. Furthermore, for some countries (Belgium, Bulgaria, Greece, Hungary, Luxembourg, Portugal, Romania, Slovakia, and Spain), cancer incidence estimates were not available in the Global Cancer Observatory (GCO) for the earlier years analyzed (2012, 2014, and 2017). Therefore, data from the Global Burden of Disease (GBD) database were used where available; however, for these countries, GBD data were available only for selected cancer types and did not provide estimates for all cancers combined. This difference in data availability and classification may limit the comparability and comprehensiveness of the analysis. Although the European clinical trials registration system was harmonized by Regulation (EU) No 536/2014 of the European Parliament and of the Council, the data used in this study come from the EU Clinical Trials Register (EUCTR) and may be incomplete or outdated, which could lead to underestimation or overestimation of research activity. A temporal mismatch between epidemiological data and clinical trials is also possible, as the latter often reflect earlier epidemiological trends. As a result, short-term fluctuations or intermediate trends between the analyzed years may not be reflected in the presented analysis, potentially limiting the precision of temporal interpretations. Counting multicenter trials separately for each country may also overestimate the level of research activity. Furthermore, differences in the number of trials between countries may result not only from disease burden but also from systemic factors such as infrastructure, funding, and regulations. The classification of trials into commercial and non-commercial was based on the type of sponsor, which may not fully reflect the complexity of funding structures. Another important limitation is that our analysis was based on the number of registered clinical trials rather than the number of patients enrolled. Because recruitment rates vary considerably across studies and some trials fail to achieve their target enrollment, the number of trials is not necessarily a reliable surrogate for the number of patients who ultimately benefit from participation in clinical research. Likewise, data on the number of patients receiving treatment within clinical trials were not consistently available and therefore could not be included in the present analysis.

## 5. Conclusions

This study shows that despite increasing clinical research activity across European Union countries, substantial disparities in research efforts regarding the burden of cancer persist. These findings indicate that current research activity is not fully aligned with public health needs and highlight the need for more sustainable and focused research development, especially non-commercial research. Non-commercial research, including international research, plays an important role because its goal is to generate reliable clinical evidence that improves the quality of healthcare, focusing not on profit but on the actual needs of patients. Therefore, future research should further explore the factors influencing these disparities and support strategies to improve the alignment of public health needs with oncology clinical research priorities.

## Figures and Tables

**Table 1:** Overall cancer incidence and incidence of breast, colon, lung and prostate cancer along with the number of commercial and non-commercial clinical trials in 2022 in European Union countries.

	All Cancers			Breast Cancer			Colorectal Cancer			Lung Cancer			Prostate Cancer		
Country	Incidence, n	Non-Comm. CTs	Comm. CTs	Incidence, n	Non-Comm. CTs	Comm. CTs	Incidence, n	Non-Comm. CTs	Comm. CTs	Incidence, n	Non-Comm. CTs	Comm. CTs	Incidence, n	Non-Comm. CTs	Comm. CTs
Austria	45,525	12	77	5983	5	19	4539	1	4	5410	2	28	5934	1	11
Belgium	72,680	28	169	11,255	10	37	7949	0	12	8636	2	58	10,523	8	18
Bulgaria	31,485	1	32	3558	0	7	5086	0	2	3966	1	12	3122	0	6
Croatia	27,512	1	8	3108	1	2	4190	0	0	3649	0	3	3247	0	0
Cyprus	5921	1	0	1020	0	0	568	0	0	625	0	0	691	1	0
Czech Republic	61,107	4	87	7732	1	13	7568	0	7	6192	0	15	7956	0	22
Denmark	43,964	24	61	5259	3	11	6878	1	8	4973	2	12	5250	2	10
Estonia	7817	2	8	857	0	0	1074	0	1	810	1	2	1174	1	0
Finland	35,274	7	35	5378	1	5	4020	1	2	3068	0	6	5930	4	8
France	435,114	78	259	65,659	22	45	51,636	2	20	49,613	16	101	57,357	6	33
Greece	63,176	1	79	8987	0	13	7859	0	6	8583	0	31	7036	0	2
Spain	264,528	48	341	34,735	16	77	39,421	2	25	30,041	10	132	32,967	7	35
Netherlands	116,224	45	124	15,919	11	13	17,670	1	9	14,806	5	57	11,956	5	21
Ireland	26,884	10	42	3688	4	11	3121	0	2	3086	0	11	4216	5	3
Lithuania	16,119	2	15	1719	0	0	1506	0	1	1494	2	5	3208	0	3
Luxembourg	2983	0	0	565	0	0	316	0	0	360	0	0	443	0	0
Latvia	11,082	0	9	1263	0	1	1127	0	0	1018	0	1	1662	0	1
Malta	2738	0	0	386	0	0	301	0	0	227	0	0	284	0	0
Germany	529,955	35	212	74,016	8	44	62,544	1	14	62,025	6	78	65,269	6	22
Poland	202,037	8	199	24,418	2	42	26,888	0	13	30,379	1	83	22,480	2	23
Portugal	66,600	4	73	8954	2	27	10,575	0	4	6155	1	27	7529	0	5
Romania	100,471	3	67	12,685	1	11	13,541	0	4	11,716	1	31	10,442	1	6
Slovakia	29,273	1	21	3649	0	6	4219	0	4	2641	0	2	3606	1	9
Slovenia	13,719	0	5	1660	0	2	1580	0	0	1729	0	3	1765	0	0
Sweden	61,794	26	64	7452	6	13	7699	0	4	4441	5	13	11,732	3	13
Hungary	62,509	3	103	7694	2	29	9607	0	3	9911	1	33	6660	0	11
Italy	407,240	52	240	57,480	13	47	54,784	2	20	43,808	3	98	38,180	6	21

CTs—clinical trials; Comm.—commercial; Non-comm.—non-commercial; incidence, n—number of newly diagnosed cancer cases in 2022.

**Table 2:** Overall cancer incidence and incidence of breast, colon, lung and prostate cancer along with the number of commercial and non-commercial clinical trials in 2017 in European Union countries.

	All Cancers			Breast Cancer			Colorectal Cancer			Lung Cancer			Prostate Cancer		
Country	Incidence, n	Non-Comm. CTs	Comm. CTs	Incidence, n	Non-Comm. CTs	Comm. CTs	Incidence, n	Non-Comm. CTs	Comm. CTs	Incidence, n	Non-Comm. CTs	Comm. CTs	Incidence, n	Non-Comm. CTs	Comm. CTs
Austria	42,223	3	8	5377	1	3	4415	0	0	4916	0	0	5782	1	4
Belgium	68,531	9	23	10,558	3	5	10,589	1	1	8974	0	10	7639	4	3
Bulgaria	-	1	3	4504	0	0	6065	0	0	4488	1	3	3968	0	0
Croatia	25,336	0	1	2771	0	0	3763	0	0	3323	0	0	2841	0	0
Cyprus	3760	0	0	621	0	0	362	0	0	360	0	0	493	0	0
Czech Republic	60,011	0	16	7058	0	2	7486	0	0	6730	0	3	7960	0	4
Denmark	39,947	5	11	4794	0	2	5459	0	0	4890	0	0	4422	1	4
Estonia	7590	0	0	767	0	0	1040	0	0	834	0	0	1134	0	0
Finland	33,208	1	6	4964	0	1	3447	0	0	2878	0	0	5468	0	3
France	44,872	15	35	6610	5	7	5159	1	0	5173	1	10	6528	3	5
Greece	-	0	8	10,070	0	2	9378	0	0	9737	0	1	7144	0	0
Spain	-	10	44	31,847	3	9	50,969	0	0	33,949	1	14	30,492	5	7
Netherlands	105,646	9	18	14,969	5	3	15,025	1	0	13,472	1	5	12,041	0	5
Ireland	23,339	6	6	3346	3	2	2697	0	0	2698	0	1	3714	3	1
Lithuania	15,302	0	1	1443	0	0	1660	0	0	1555	0	0	2272	0	0
Luxembourg	-	0	0	462	0	0	477	0	0	349	0	0	311	0	0
Latvia	10,373	0	0	1157	0	0	1128	0	0	1128	0	0	1334	0	0
Malta	2120	0	0	337	0	0	257	0	0	215	0	0	179	0	0
Germany	41,698	2	27	5812	1	6	5013	0	0	5083	0	9	4989	1	6
Poland	5421	3	31	538	1	6	659	0	0	736	1	11	717	1	5
Portugal	-	1	9	10,784	1	5	14,556	0	0	5953	0	2	10,835	0	0
Romania	-	1	12	11,318	0	1	12,605	0	0	11,908	1	4	6513	0	4
Slovakia	-	0	3	3200	0	0	4241	0	0	3077	0	0	2260	0	3
Slovenia	11,927	0	0	1416	0	0	1351	0	0	1474	0	0	1629	0	0
Sweden	55,772	6	15	7435	3	5	6949	1	0	4328	0	1	10,431	0	4
Hungary	-	2	10	6498	1	4	9544	0	0	9772	1	2	4355	0	0
Italy	14,718	10	33	2071	2	9	1782	1	1	1610	2	11	1516	3	5

CTs—clinical trials; Comm.—commercial; Non-comm.—non-commercial; incidence, n—number of newly diagnosed cancer cases in 2017.

**Table 3:** Overall cancer incidence and incidence of breast, colon, lung and prostate cancer along with the number of commercial and non-commercial clinical trials in 2014 in European Union countries.

	All Cancers			Breast Cancer			Colorectal Cancer			Lung Cancer			Prostate Cancer			
Country	Incidence, n	Non-Comm. CTs	Comm. CTs	Incidence, n	Non-Comm. CTs	Comm. CTs	Incidence, n	Non-Comm. CTs	Comm. CTs	Incidence, n	Non-Comm. CTs	Comm. CTs	Incidence, n	Non-Comm. CTs	Comm. CTs	
Austria	41,178	0	2	5612	0	1	4863	0	0	4971	0	0	4718	0	1	41,178
Belgium	-	3	6	10,898	2	2	10,544	0	1	9539	0	1	7339	1	1	-
Bulgaria	-	0	0	4594	0	0	5928	0	0	4505	0	0	3837	0	0	-
Croatia	23,877	0	0	2795	0	0	3665	0	0	3241	0	0	1967	0	0	23,877
Cyprus	3538	0	0	569	0	0	377	0	0	323	0	0	448	0	0	3538
Czech Republic	58,935	0	1	6968	0	0	8435	0	0	6487	0	0	6733	0	1	58,935
Denmark	39,336	1	1	4769	0	0	5585	0	0	4726	0	0	4691	0	1	39,336
Estonia	7338	0	0	736	0	0	921	0	0	831	0	0	1109	0	0	7338
Finland	30,974	0	0	4816	0	0	3198	0	0	2744	0	0	4702	0	0	30,974
France	42,196	7	5	6551	3	2	4823	0	0	4760	0	0	5673	1	1	42,196
Greece	-	0	1	9598	0	1	9090	0	0	9652	0	0	7245	0	0	-
Spain	-	2	8	31,105	1	3	50,973	0	0	33,037	0	2	30,791	1	1	-
Netherlands	100,027	1	3	14,651	1	1	15,726	0	0	12,521	0	0	10,238	0	1	100,027
Ireland	21,600	2	0	2915	1	0	2582	0	0	2451	0	0	3455	1	0	21,600
Lithuania	16,036	0	0	1673	0	0	1577	0	0	1479	0	0	3243	0	0	16,036
Luxembourg	-	0	0	9893	0	0	14,479	0	0	5714	0	0	10,225	0	0	-
Latvia	10,250	0	0	1169	0	0	1150	0	0	1098	0	0	1197	0	0	10,250
Malta	2085	0	0	316	0	0	268	0	0	192	0	0	194	0	0	2085
Germany	41,696	0	4	6013	0	2	5175	0	2	5165	0	0	4717	0	1	41,696
Poland	5099	0	6	515	0	2	630	0	0	838	0	1	488	0	1	5099
Portugal	-	0	3	9893	0	1	14,479	0	0	5714	0	1	10,225	0	0	-
Romania	-	0	1	10,619	0	0	11,607	0	0	11,823	0	0	6268	0	1	-
Slovakia	-	0	1	2952	0	0	3942	0	0	3128	0	0	2008	0	1	-
Slovenia	11,515	0	0	1305	0	0	1443	0	0	1331	0	0	1512	0	0	11,515
Sweden	54,501	2	2	7527	2	1	6291	0	0	4133	0	0	11,153	0	0	54,501
Hungary	-	0	1	6403	0	1	9535	0	0	9670	0	0	4086	0	0	-
Italy	14,358	0	6	1994	0	2	1886	0	0	1521	0	1	1400	0	1	14,358

CTs—clinical trials; Comm.—commercial; Non-comm.—non-commercial; incidence, n—number of newly diagnosed cancer cases in 2014.

**Table 4:** Overall cancer incidence and incidence of breast, colon, lung and prostate cancer along with the number of commercial and non-commercial clinical trials in 2012 in European Union countries.

	All Cancers			Breast Cancer			Colorectal Cancer			Lung Cancer			Prostate Cancer			
Country	Incidence, n	Non-Comm. CTs	Comm. CTs	Incidence, n	Non-Comm. CTs	Comm. CTs	Incidence, n	Non-Comm. CTs	Comm. CTs	Incidence, n	Non-Comm. CTs	Comm. CTs	Incidence, n	Non-Comm. CTs	Comm. CTs	
Austria	40,370	0	0	5608	0	0	4841	0	0	4773	0	0	4676	0	0	40,370
Belgium	-	1	1	11,082	1	0	10,899	0	0	9361	0	0	7263	0	0	-
Bulgaria	-	0	0	4418	0	0	5762	0	0	4576	0	0	3591	0	0	-
Croatia	22,258	0	0	2594	0	0	3178	0	0	2991	0	0	1864	0	0	22,258
Cyprus	3159	0	0	501	0	0	379	0	0	283	0	0	467	0	0	3159
Czech Republic	57,771	0	0	6831	0	0	7947	0	0	6585	0	0	7019	0	0	57,771
Denmark	36,710	0	0	4555	0	0	4549	0	0	4623	0	0	4422	0	0	36,710
Estonia	7203	0	0	724	0	0	884	0	0	817	0	0	1176	0	0	7203
Finland	29,194	0	0	4441	0	0	2984	0	0	2508	0	0	4668	0	0	29,194
France	40,830	2	1	6103	1	0	4682	0	0	4602	0	0	5608	0	0	40,830
Greece	-	0	0	9386	0	0	8610	0	0	9259	0	0	6623	0	0	-
Spain	42,993	1	1	4971	1	0	6576	0	0	4555	0	0	5433	0	0	42,993
Netherlands	94,594	0	0	14,645	0	0	13,759	0	0	12,066	0	0	11,269	0	0	94,594
Ireland	20,763	1	0	2879	1	0	2575	0	0	2376	0	0	3469	0	0	20,763
Lithuania	15,827	0	0	1519	0	0	1699	0	0	1459	0	0	2631	0	0	15,827
Luxembourg	-	0	0	446	0	0	487	0	0	334	0	0	297	0	0	-
Latvia	9974	0	0	1094	0	0	1246	0	0	1112	0	0	1081	0	0	9974
Malta	1857	0	0	302	0	0	229	0	0	202	0	0	163	0	0	1857
Germany	39,773	0	0	5967	0	0	4861	0	0	4840	0	0	4731	0	0	39,773
Poland	4865	0	1	489	0	0	618	0	0	735	0	0	445	0	0	4865
Portugal	-	0	1	10,553	0	0	14,607	0	0	5607	0	0	10,679	0	0	-
Romania	-	0	0	10,007	0	0	10,908	0	0	11,291	0	0	5803	0	0	-
Slovakia	-	0	0	2800	0	0	3750	0	0	2984	0	0	1856	0	0	-
Slovenia	11,245	0	0	1319	0	0	1555	0	0	1272	0	0	1510	0	0	11,245
Sweden	49,613	0	1	7057	0	0	6161	0	0	3941	0	0	9156	0	0	49,613
Hungary	-	0	0	6402	0	0	9626	0	0	9661	0	0	3728	0	0	-
Italy	14,234	0	0	2001	0	0	1903	0	0	1493	0	0	1524	0	0	14,234

CTs—clinical trials; Comm.—commercial; Non-comm.—non-commercial; incidence, n—number of newly diagnosed cancer cases in 2012.

**Table 5:** Pearson and Spearman correlations between cancer incidence and commercial and non-commercial clinical trial activity in European Union countries in 2014, 2017, and 2022, with 95% confidence intervals and *p*-values.

Year	Cancer Type	Commercial CTs						Non-Commercial CTs					
		r	95% CI	*p*	ρ	95% CI	*p*	r	95% CI	*p*	ρ	95% CI	*p*
2022	Breast cancer	0.760	0.534–0.885	<0.001	0.929	0.848–0.967	<0.001	0.770	0.552–0.890	<0.001	0.800	0.603–0.905	<0.001
	Colorectal cancer	0.865	0.723–0.937	<0.001	0.902	0.794–0.955	<0.001	0.753	0.523–0.881	<0.001	0.529	0.187–0.757	0.005
	Lung cancer	0.853	0.701–0.931	<0.001	0.971	0.937–0.987	<0.001	0.704	0.443–0.855	<0.001	0.676	0.398–0.840	<0.001
	Prostate cancer	0.771	0.552–0.890	<0.001	0.890	0.770–0.949	<0.001	0.669	0.388–0.837	<0.001	0.666	0.383–0.835	<0.001
2017	Breast cancer	0.528	0.185–0.756	0.005	0.582	0.260–0.788	0.001	0.483	0.126–0.729	0.011	0.504	0.154–0.742	0.007
	Colorectal cancer	0.021	−0.398–0.362	0.918	0.073	−0.316–0.440	0.719	0.050	−0.336–0.422	0.805	0.282	−0.110–0.598	0.155
	Lung cancer	0.519	0.174–0.751	0.005	0.563	0.233–0.777	0.002	0.338	−0.049–0.636	0.085	0.353	−0.031–0.646	0.071
	Prostate cancer	0.525	0.181–0.754	0.005	0.465	0.103–0.718	0.015	0.507	0.157–0.744	0.007	0.207	−0.188–0.544	0.301
2014	Breast cancer	0.546	0.210–0.767	0.003	0.423	0.052–0.692	0.028	0.335	−0.052–0.634	0.088	0.472	0.113–0.723	0.013
	Colorectal cancer	0.011	−0.390–0.370	0.955	0.159	−0.236–0.508	0.429	-	-	-	-	-	-
	Lung cancer	0.584	0.263–0.789	0.001	0.153	−0.241–0.504	0.447	-	-	-	-	-	-
	Prostate cancer	0.235	−0.160–0.564	0.239	0.268	−0.125–0.588	0.177	0.444	0.077–0.705	0.020	0.295	−0.096–0.607	0.136

CTs, clinical trials; CI, confidence interval; r, Pearson correlation coefficient; ρ, Spearman rank correlation coefficient; *p*, *p*-value. Pearson coefficients assess linear relationships, whereas Spearman coefficients assess monotonic rank-based relationships. “-” indicates that the analysis was not applicable.

## Data Availability

The data analyzed in this study were derived from publicly available sources, including the Clinical Trials Information System public website (https://euclinicaltrials.eu/), the Global Cancer Observatory (https://gco.iarc.who.int/), the Global Burden of Disease database https://vizhub.healthdata.org/gbd-compare/ (accessed on 2 June 2026), and Eurostat https://ec.europa.eu/eurostat (accessed on 2 June 2026). The data supporting the findings of this study are included in the article. Further inquiries can be directed to the corresponding author.
